# Different effects of soybean isoflavone genistein on transforming growth factor levels during orthodontic tooth movement among young and old rabbits

**DOI:** 10.12688/f1000research.21211.2

**Published:** 2020-06-04

**Authors:** Verastuti Indriasari, Sri Suparwitri, Christnawati Christnawati, Ananto Ali Alhasyimi

**Affiliations:** 1 Department of Orthodontics, Faculty of Dentistry, Universitas Gadjah Mada, Sleman, Special Region of Yogyakarta, 55281, Indonesia

**Keywords:** genistein, juvenile, adult, orthodontic tooth movement

## Abstract

**Background: **Orthodontic treatment to improve aesthetics and for health reasons is performed in children and adults. Elderly individuals have low levels of estrogen, this results in alveolar bone resorption being greater than alveolar bone apposition. Isoflavones present in soybeans may be able to improve the remodeling process through the induction of osteoblastogenesis by increasing transforming growth factor-β1 (TGF-β1) levels. This study aimed to assess the comparative effect of soybean genistein isoflavone to TGF-β1 during orthodontic tooth movement among juvenile and adult rabbits.

**Method** **s**: In this study, 12 healthy female rabbits were used. Subjects were divided into four groups (n=3); YG group (young rabbits), YGI group (young rabbits + isoflavones genistein), OG group (old rabbits), and OGI group (old rabbits + isoflavones genistein). Two lower incisors of the rabbit were moved distally using an orthodontic force (50 grams force) delivered by an open coil spring, which was inserted between two brackets. During active movements, the genistein isoflavones were given from the initial installation of the device until days 21, at a dose of 1.2 mg/kg BW once a day. Measurement of TGF-β levels were performed on days 1, 7, 14, 21 after appliance installation. TGF-β1 expression was analyzed using enzyme-linked immunosorbent assay (ELISA) and the optical density (OD) of the sample quantifed using a standard curve. The data obtained were analyzed using one-way Anova followed by Tukey HSD test.

**Results: **The TGF-β1 levels were found to highest in the YGI group, and the TGF-β levels were significantly lower in the OG group ( *p*<0.05). ELISA analysis also revealed that TGF-β1 levels of the OGI group were significantly higher when compared with the OG group ( *p*<0.05).

**Conclusion:** The administration of soybean genistein isoflavones could improve TGF-β1 levels in old rabbit’s during active orthodontic tooth movement.

## Introduction

Today’s society is witnessing an increased interest in cosmetic dentistry, thereby making orthodontics a necessity ^1^. Orthodontic treatment has become one of the most procedures in cosmetic dentistry; it is performed to improve malocclusion, achieve good occlusion and dentofacial harmony ^2, 3^. The demand for orthodontic treatment has grown over time, not only in children, but also in adults ^4^. The rising number of adults demanding orthodontic treatment presents a new challenge. A previous study found that adults alveolar bone remodeling is much slower than in juveniles due to decreased cellular activity and vascularity, this suggests that orthodontic treatment duration may be longer in adults due to a delay in orthodontic tooth movement (OTM) ^5^.

The success of orthodontic treatment depends on the process of alveolar bone tissue remodeling during the treatment which involves bone apposition and resorption by osteoblasts and osteoclasts ^6^. For children and juveniles who are in an early stage of growth and development, bone apposition and resorption are balanced. In contrast, in adults, and following the menopause, the rate of bone apposition is less than that of resorption ^7^. With increasing age, estrogen levels decrease, resulting an increased incidence of osteoporosis with associated complications ^8^. In the context of adult orthodontic patients, it may be beneficial to address this deficiency to improve treatment outcome.

Hughes *et al.* ^9^, demonstrated that estrogen replacement may help to inhibit excessive bone loss by restricting osteoclast life span through promotion of apoptosis, mediated by transforming growth factor beta (TGF-β1). Estrogen effectively modulates TGF-β1 production in osteoblast and other cells. TGF-β1 is one of the most significant factors in bone formation, helping to maintain the balance between the dynamic processes of bone formation and bone resorption ^10^. The development of natural remedies for the promotion of this mechanism, specifically, could be a useful and novel therapeutic approach to enhance bone remodeling by modulating the levels of TGF-β1 in adults during OTM.

Nowadays, the use of hormones derived from natural ingredients, namely phytohormones, has gained a lot of popularity. One of these is phytoestrogen, a substrate from plants with estrogen-like activity. The isoflavone genistein is a type of phytoestrogen which is the main polyphenol component of the soybean ^11^. Some derivatives of soybeans have been recognized to have a positive effect on bone remodeling without triggering side effects. The effects of isoflavones on the improvement of osteoblast’s proliferation have previously been observed. Genistein administration has shown potential to increase osteoblast numbers during OTM ^4^. Further study to explore the effect of genistein in enhancing osteoblastogenesis through improving TGF-β1 level is needed to validate its advantage in orthodontic bone remodeling. This study aimed establish the comparative effect of genistein on TGF-β1 during orthodontic tooth movement among juvenile and adult rabbits. The hypothesis of this study is that the administration of soybean genistein isoflavones could improve TGF-β1 levels during active orthodontic tooth movement, espescially in old rabbit. The rabbits were selected as a model as it they have previously used to study the effect of medications on OTM ^12^. Rabbit also provide an excellent model system to simulate the response of human tissue and are not aggressive, making them easy to handle and observe ^13^.

## Methods

### Animal study

All experimental procedures involving animals were carried out in keeping with guidelines from the National Institutes of Health Guide for the Care and Use of Laboratory Animals to ameliorate any suffering of animals. Ethical clearance was obtained from the Research Ethics Committee of the Faculty of Dentistry, Universitas Gadjah Mada, Indonesia, with number 00242/KKEP/FKG-UGM/EC/2019.

We used 12 female rabbits ( *Oryctolagus cuniculus*) (Integrated Laboratory of Research and Testing, UGM, Indonesia), which were randomly divided into 4 groups (n=3), YG group (young rabbits/controls), YGI (young rabbits + isoflavones genistein), OG (old rabbits/controls), OGI (old rabbits + isoflavones genistein). Young rabbits were 3 months old and weighed approximately 1000 grams, whilst old rabbits were 3 years old and weighed approximately 4000 grams. Sample size (n=3) was determine based on Lemeshow's formula. A sample size of three animals in each group would present more than 85% power to detect significant differences with 0.45 effect size and at a significance level of α= .05. Sample groups were chosen utilizing simple random sampling. Each animal was assigned a tag number, the blind-folded researcher then picks numbered tags from the hat.

All the rabbits were housed individually in polycarbonate cages (0.90 × 0.60 × 0.60 m) for a week on a 12-h light/dark cycle at a steady temperature of 25°C and humidity of 50% for acclimatization to compensate for their various origins. Animals were fed a standard pellet diet with tap water *ad libitum,* and were routinely inspected for food consumption and fecal characteristics.

**Figure 1.**
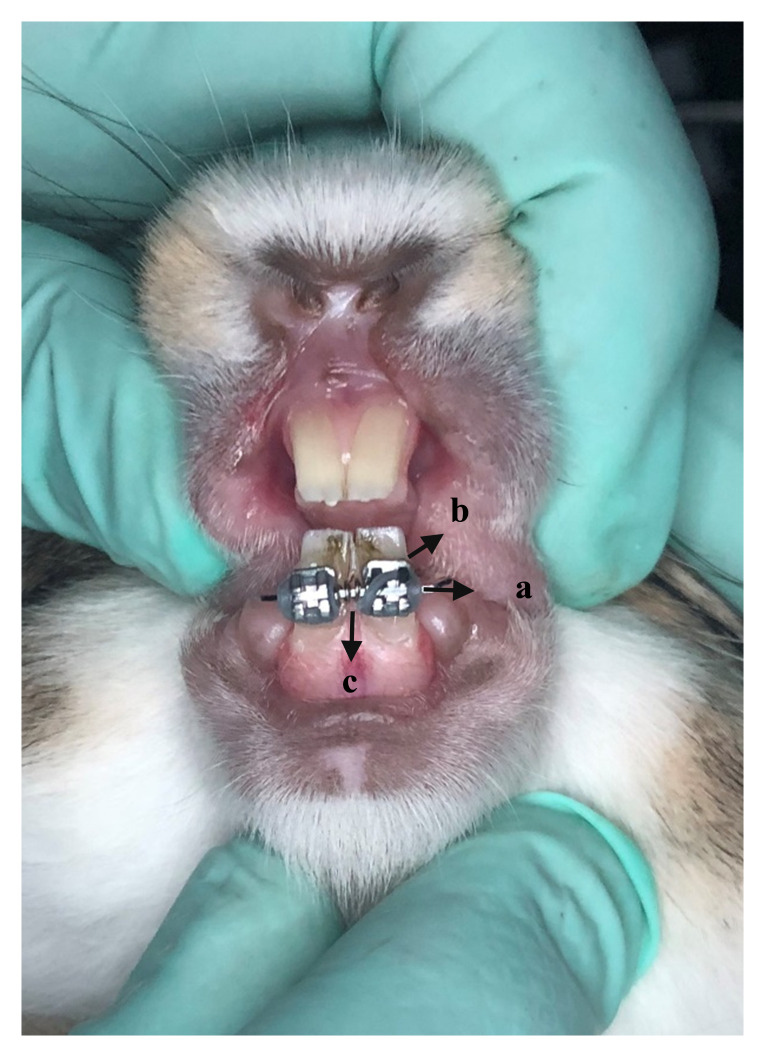
Design of the orthodontic device: ( **a**) rectangular stainless steel wire, ( **b**) preadjusted edgewise bracket, and ( **c**) open coil spring.

Prior to preforming the experimental procedure, rabbits were anesthetized with ketamine (160095, Kepro™, Netherlands), and xylazine (160096, Xyla™, Netherlands) (ketamine dose 35 mg/kg body weight and xylazine 5 mg/kg body weight), intramuscularly on the gluteus muscle during installation of the orthodontic appliance in their mouth. Two lower incisors of the rabbit were moved distally using a NiTi open coil spring 0.010”x 0.030” (O-951-1200, DynaFlex, the Netherlands) which was inserted between two preadjusted edgewise lower incisor brackets engaged to a 0.016 "x 0.016" rectangular stainless-steel wire (126-029977, American Orthodontics®, USA) ( Figure 1). An open coil spring was compressed until it produced 50 grams orthodontic force continuously for 21 days (measured by tension gauge, MedKraft Orthodontics, USA). No reactivation of the appliance was done throughout the experiment. During active tooth movements, the genistein (produced by Prof. Mien Karmini from IPB) were given from the initial installation of the device until days 1, 7, 14 and 21, at a dose of 1.2 mg/kg BW dissolved in 5 ml of distilled water. This dose used was determined from evidence from a previous *in vivo* study ^14^.

The composition of genistein consists of tempeh, wheat flour, sugar, salt and vegetable oil. The processing process is as follows. Fresh tempeh first cut it into a kind of dice, then boiled in boiling water for 10 minutes. After draining, the tempe is ground with a grinder meat. Tempe that has been ground and then mixed in a mixer that has been light with a little water and all the other ingredients that have been prepared, then wait for 15 minutes or until the dough becomes smooth. Batter it is then flattened in a pan, pressed and made lines as an air cavity. The dough is then baked in the oven with a temperature of 175° up to 200°C for 10 minutes. After that the dough has been shaped like a biscuit is cut into small pieces and then dried use a dryer with a temperature of 70°C for about 16 hours. After dried, the tempe cereal is ground with a kind of flour grinder until it becomes powder.

The genistein solution was then given orally using a nasogastric tube once a day. Before gingival crevicular fluid sample collection, all the animals were observed for any general toxicity probability, including edema or deaths, and measured the body weight (using a digital scale, ZB22-P, Zieis®, USA). All these measurements were done by a single blinded observer.

### Isolation of gingival crevicular fluid (GCF)

**Figure 2.**
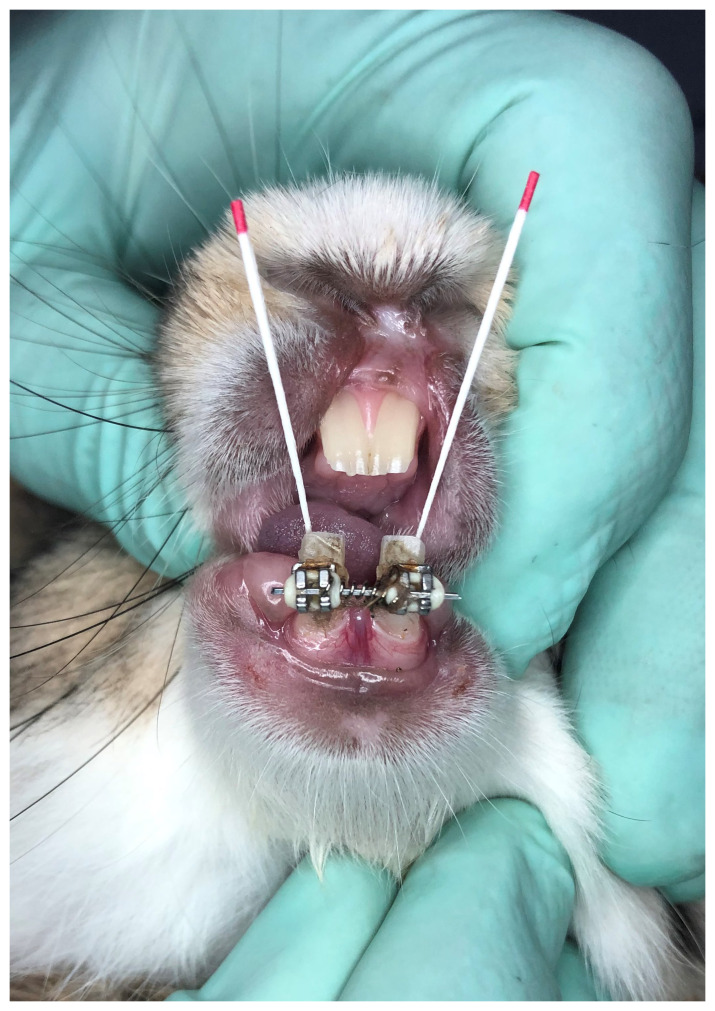
Gingival crevicular fluid isolation.

The GCF samples were collected from the two interproximal areas of the maxillary anterior teeth (mesial and distal sides) at four subsequent time points (1, 4, 7, and 14 days after the installation of the orthodontic appliance) ( Figure 2). During GCF collection all animals were sedated. Two #15 sterilized paper points (A-022T, Dentsply, Germany) were used to collect the GCF. The paper points were gently inserted approximately 1 mm into the gingival sulcus and were left *in situ* for 30 s after removing the supragingival plaque with cotton swabs. Thereafter, the paper points were isolated with cotton rolls and dried. The dipped paper points were then stored in a sterile 1.5 ml tube comprising 350 µl of physiological saline solution (Nova-Tech, Inc., USA). The tube was centrifuged at 2000 rpm for 5 min at 4°C with the help of a microcentrifuge refrigerator (Eppendorf 5424R, USA) to elute the entire GCF element from the paper points. Paper points were removed, and the supernatant solution was kept at a temperature of −80°C in a refrigerator until further analysis. After the collection of the final samples, all rabbits were sacrificed with an overdosed anesthesia (intravenous injection of 100 mg/kg BW Pentobarbital, 1507002, Pubchem, USA) following Guidelines for the Euthanasia of Animals by American Veterinary Medical Association ^15^ to collect bone samples for further histological analysis (Results not reported).

The TGF-β1 levels were detected and analyzed by enzyme-linked immunosorbent assay (ELISA). The analysis was done using a quantitative sandwich Rabbit TGF-β1 ELISA kit (ERB0119, FineTest, China). The TGF-β1 levels in the sample were found by extrapolating the optical densities of the samples on the standard curve. The optical densities were measured at 450 nm using a microplate reader (Bio-Rad Laboratories Inc., USA), single readings were taken for each animal. TGF-β1 expression levels were calculated as pg/mL. One-way ANOVA tests followed by Tukey’s *post-hoc* test were used to identify possible differences in TGF-β1 expression between controls and treatment groups. Statistical significance was set at *p* values < 0.05. Statistical analysis was processed with the SPSS 21.0 software system (SPSS Inc., Chicago, Illinois, USA).

## Results

In general, giving genistein isoflavones at the selected dose did not cause any general toxicity, edema or deaths, nor did it affect the body weight of rabbits (see underlying data ^16^). ELISA analysis revealed that in young rabbits with OTM receiving genistein, TGF-β1 levels were significantly higher than other groups on day 1, 7, 14 and 21 ( *p* < 0.05). In older rabbits with OTM receiving genistein, TGF-β1 levels were almost the same as those in young rabbits without genistein isoflavones administration on days 1, 7, 14 and 21 ( *p* > 0.05). In the old rabbits group without genistein administration, TGF-β1 levels were significantly lower than the other groups on days 1, 7, 14 and 21 after bonding ( *p* < 0.05). The YG and OGI group showed no significant difference in TGF-β1 levels at all time points ( *p* > 0.05) ( Table 1– Table 4; underlying data ^17^).

**Table 1.** Descriptive statistics and results of the Anova and Tukey HSD tests comparing the TGF-β1 in the 4 groups tested at day 1 after orthodontic appliance installation.

Group	N	TGF-β1 level (pg/ml)	Significance *	*p-*value		
				YGI	OG	OGI
**YG**	3	40.34±3.22	*P*= 0.037 *	0.228	0.001 *	0.763
**YGI**	3	49.03±4.02			0.000 *	0.058
**OG**	3	14.44±3.09				0.003 *
**OGI**	3	36.31±3.89				

Values are presented as mean ± standard deviation or *p*-value only. *by ANOVA, *Significant differences between groups ( *p* < 0.05). ANOVA: Analysis of variance; YG: young rabbit, YGI: young rabbits+isoflavones genistein, OG: old rabbit, OGI: old rabbits+isoflavones genistein).

**Table 2.** Descriptive statistics and results of the Anova and Tukey HSD tests comparing the TGF-β1 in the 4 groups tested at day 7 after orthodontic appliance installation.

Group	N	TGF-β1 level (pg/ml)	Significance *	*p-*value		
				YGI	OG	OGI
**YG**	3	31.44±3.92	*P*= 0.001 *	0.998	0.001 *	0.098
**YGI**	3	31.95±2.64			0.001 *	0.075
**OG**	3	15.06±1.22				0.044 *
**OGI**	3	24.03±2.42				

Values are presented as mean ± standard deviation or p-value only. *by ANOVA, *Significant differences between groups ( *p* < 0.05) ANOVA: Analysis of variance; YG: young rabbit, YGI: young rabbits+isoflavones genistein, OG: old rabbit, OGI: old rabbits+isoflavones genistein).

**Table 3.** Descriptive statistics and results of the Anova and Tukey HSD tests comparing the TGF-β1 in the 4 groups tested at day 14 after orthodontic appliance installation.

Group	N	TGF-β1 level (pg/ml)	Significance *	*p-*value		
				YGI	OG	OGI
**YG**	3	40.69.±3.25	*P=* 0.000 *	0.634	0.000 *	1.000
**YGI**	3	44.85±4.63			0.000 *	0.590
**OG**	3	15.15±3.07				0.000 *
**OGI**	3	40.43±2.26				

Values are presented as mean ± standard deviation or *p*-value only. *by ANOVA, *Significant differences between groups ( *p* < 0.05). ANOVA: Analysis of variance; YG: young rabbit, YGI: young rabbits+isoflavones genistein, OG: old rabbit, OGI: old rabbits+isoflavones genistein).

**Table 4.** Descriptive statistics and results of the Anova and Tukey HSD tests comparing the TGF-β1 in the 4 groups tested at day 21 after orthodontic appliance installation.

Group	N	TGF-β1 level (pg/ml)	Significance *	*p-*value		
				YGI	OG	OGI
**YG**	3	27.21.±6.47	*P=* 0.045 *	1.000	0.067	0.984
**YGI**	3	27.53±4.98			0.048 *	0.973
**OG**	3	12.63±2.76				0.042 *
**OGI**	3	25.49±4.52				

Values are presented as mean ± standard deviation or *p*-value only. *by ANOVA, *Significant differences between groups ( *p* < 0.05). ANOVA: Analysis of variance; YG: young rabbit, YGI: young rabbits+isoflavones genistein, OG: old rabbit, OGI: old rabbits+isoflavones genistein).

## Discussion

This investigation confirms the hypothesis that soybean isoflavone genistein administration could increase the TGF-β1 levels during orthodontic tooth movement, especially in older rabbits. In general, results demonstrated that in the subjects receiving soybean isoflavone genistein, TGF-β1 levels were significantly higher than the other groups. A previous study found that genistein can significantly increase the number of osteoblasts during orthodontic tooth movement ^4^. Soybeans are the most common source of isoflavones, which have physiological effects that mimic native estrogen in maintaining bone formation rates in rats after ovariectomy-induced osteoporosis ^18^. A previous *in vitro* study reported that genistein increased alkaline phosphatase expression along with protein and DNA content in osteoblastic MC3T3-E1 cells, indicating an anabolic effect ^19^. Osteoblastogenesis is induced by TGF-β1, which is a physiological regulator of osteoblast differentiation and acts as a central component of the coupling of bone formation to resorption during bone remodeling ^20^. TGF-β1 is osteogenic growth factor that has highly osteogenic attributes, enhancing osteoblast activity (by inducing bone marrow mesenchymal stem cells to differentiate into osteoblasts), stimulating osteoclast apoptosis, restraining osteoclastic activity, and resulting in bone formation ^21^. Moreover, TGF-β1 has been found to perform a critical role in tissue regeneration as it potently improves the synthesis of connective tissue elements, such as type I collagen, proteoglycans, osteopontin, fibronectin, and osteonectin, during alveolar bone remodeling. The TGF-β1 superfamily further includes bone morphogenetic proteins (BMPs), which are the most potent inducers of bone formation ^22^.

Our results showed that TGF-β1 levels in old rabbits are lower than young rabbits. Aging can cause a decreasing in the osteoblasts number due to an imbalance in bone remodeling ^23^. Impaired osteoblastogenesis induced by maturation will cause a characteristic of bone loss. It is widely accepted that the loss of bone with aging is a universal phenomenon which is associated with reduced bone strength. In adult individuals, the microarchitecture of trabecular bone becomes thin, while cortical bone becomes thin and porous ^24^. A previous *in vivo* study demonstrated that in response to mechanical stimuli, the alveolar bone of the young rats was more active and sensitive than that of the adult rats ^4^. As the aging process progresses, the periodontal ligament becomes more fibrotic and may influence how the tissues react to orthodontic forces ^25^.

The old rabbit group exposed to soybean isoflavone genistein exhibited higher TGF-β1 levels than the old rabbit’s groups without soybean isoflavone genistein and this value was almost similar to the young rabbit’s groups without soybean isoflavone genistein. This is caused by administration of genistein to old rabbit’s groups which can overcome bone formation problems that are caused by a decrease in estrogen. Genistein is a plant compound with potent estrogenic activity that has similarity in structure with the human female hormone 17-β-estradiol. This hormone can bind to both alpha and beta estrogen receptors, and imitate the action of estrogens on target organs, and thereby it can provide many health benefits when used in some hormone-dependent conditions, including old age ^26^. Estrogen prevents loss of bone mineral density through a TGFβ-dependent mechanism, which stimulate TGF-β1 production in the bone marrow with a critical “upstream” mechanism. The main role of TGF-β1 in osteoblastogenesis and bone formation is in recruiting osteoblast progenitors, stimulating their proliferation (increased DNA synthesis) and promoting the early stages of differentiation (bone matrix production) to increases bone formation. In addition, apoptosis of osteoblasts is blocked through maintenance of survival during transdifferentiation into osteocytes by TGF-β1.

High levels of TGF-β1 will suppress receptor activator of nuclear factor kappa- *β* ligand (RANKL) expression, which plays a role in osteoclast differentiation as a osteoclast differentiation factors. In other words, indirectly TGF-β1 can limit the formation and activation of osteoclasts and increase bone mass ^27, 28^. This condition is expected during active orthodontic tooth movement especially in adult.

A limitation of this study was the time points of evaluation limits in 21 days during orthodontic tooth movement. Further studies evaluating alveolar bone and underlying periodontal tissue changes after genistein administration over time during orthodontic tooth movement could further illuminate the underlying biologic processes. Future studies are also needed to confirm the effective dose of material used when applied for humans.

## Conclusions

Taken together, the results of this preclinical study suggest that administration of the soybean isoflavone genistein could induce TGF-β1 levels during orthodontic tooth movement especially in old rabbits.

## Data availability

### Underlying data

Figshare: Raw Data TGF beta isoflavone genistein. https://doi.org/10.6084/m9.figshare.10116704 ^17^

This project contains the following underlying data:

- TGF LOCKY ANAVA.xlsx (Recorded TGF beta levels) ****

Figshare: OUTPUT Statistic Isoflavon TGF.docx. https://doi.org/10.6084/m9.figshare.10117340 ^29^

This project contains the following underlying data:

- OUTPUT Statistic Isoflavon TGF.docx (Output file from statistical analysis) ****

Figshare: Animal Body Weight of Rabbit. https://doi.org/10.6084/m9.figshare.10310696 ^16^

This project contains the following underlying data:

- Animal Body Weight.xlsx (Animal body weights)

Data are available under the terms of the Creative Commons Attribution 4.0 International license (CC-BY 4.0).
