# Supplementary material for: Different effects of soybean isoflavone genistein on transforming growth factor levels during orthodontic tooth movement among young and old rabbits
Source: F1000Res. 2020 Jun 4;8:2074. Originally published 2019 Dec 10. [Version 2] doi: 10.12688/f1000research.21211.2 (PMC7295133; doi:10.12688/f1000research.21211.2)
Supplement: Peer review history

# Peer review: Reviewer response for version 2

**DOI:** 10.5256/f1000research.27030.r64307

**Published:** 2020-06-12

**Authors:** Lidia Audrey Rocha Valadas

**Affiliations:** 1 Postgraduate Program in Development and Technological Innovation in Drugs, School of Pharmacy, Federal University of Ceará, Fortaleza, Brazil

Is the work clearly and accurately presented and does it cite the current literature?

Yes

If applicable, is the statistical analysis and its interpretation appropriate?

Yes

Are all the source data underlying the results available to ensure full reproducibility?

Partly

Is the study design appropriate and is the work technically sound?

Yes

Are the conclusions drawn adequately supported by the results?

Yes

Are sufficient details of methods and analysis provided to allow replication by others?

Partly

Reviewer Expertise:

NA

I confirm that I have read this submission and believe that I have an appropriate level of expertise to confirm that it is of an acceptable scientific standard.

# Peer review: Reviewer response for version 1

**DOI:** 10.5256/f1000research.23351.r62285

**Published:** 2020-05-29

**Authors:** Lidia Audrey Rocha Valadas

**Affiliations:** 1 Postgraduate Program in Development and Technological Innovation in Drugs, School of Pharmacy, Federal University of Ceará, Fortaleza, Brazil

That´s a well designed study and I have written some considerations below:

 The introduction is clear and well written;

 On the methods session I miss details of how you obtained the isoflavone, for me is the most important part to assure the reproducibility of the study.

 The discussion is small and is necessary to improve it and should add the limitations of the study.

Is the work clearly and accurately presented and does it cite the current literature?

Yes

If applicable, is the statistical analysis and its interpretation appropriate?

Yes

Are all the source data underlying the results available to ensure full reproducibility?

Partly

Is the study design appropriate and is the work technically sound?

Yes

Are the conclusions drawn adequately supported by the results?

Yes

Are sufficient details of methods and analysis provided to allow replication by others?

Partly

Reviewer Expertise:

Pharmacology and Dentistry

I confirm that I have read this submission and believe that I have an appropriate level of expertise to confirm that it is of an acceptable scientific standard, however I have significant reservations, as outlined above.

# Response

**Published:** 2020-05-29

**Authors:** Ananto Ali Alhasyimi

**Affiliations:** Universitas Gadjah Mada, Indonesia

Dear Prof **Lidia Audrey Rocha Valadas**

Thank you for your kind assistance in reviewing our manuscript and providing us with valuable advice for the manuscript and our future work. We have been working throughout the review. The following are our consolidated comments related to the review and some changes we have done based on the reviewer comments.

That´s a well designed study and I have written some considerations below:

The introduction is clear and well written;

Thank you so much

On the methods session I miss details of how you obtained the isoflavone, for me is the most important part to assure the reproducibility of the study.

We have edited the methods section and added this information in the present manuscript.

The discussion is small and is necessary to improve it and should add the limitations of the study.

We have edited the discussion section and added other references and limitation of the study as well. Thank you for the punctiliously review. We have added this information in the present manuscript

We thank you again for the valuable insight.

# Peer review: Reviewer response for version 1

**DOI:** 10.5256/f1000research.23351.r58276

**Published:** 2020-01-20

**Authors:** Ida Bagus Narmada, Alexander Patera Nugraha

**Affiliations:** 1 Department of Orthodontics, Faculty of Dental Medicine, Airlangga University, Surabaya, Indonesia; 2 Department of Orthodontics, Faculty of Dental Medicine, Airlangga University, Surabaya, Indonesia

First of all, please allow me to congratulate the authors for attempting to undertake this project which I found very interesting and of valuable additional knowledge. The manuscript itself is well-written and well-structured and I have to also commend the authors for this matter.

 However, I may require some clarifications on the following issues:

1. Paper is well organized and easy to follow.
2. Novelty is sufficient and high impact.
3. To improve the readability, It is recommended that the text is checked by a native English speaking person as many of the sentences might be misunderstood. I suggest a revision of the grammar structures by an expert editor in revising manuscripts.

Abstract:

1. Please add the p value used for ANOVA and Tukey HSD (p<0.05) add homogenity and normality test p-value used (p>0.05) in the abstract section
2. Please add the exact p value obtained after the statistic analysis. (p=.....).
3. There is a minor mistake in keyword: orthodontic orthodontic tooth movement. It would be better to choose the keyword according the MeSH.

Introduction:

1. Please add your hypothesis
2. Please add the statement or sentence about is there any similar studies have been done before or not? or your study is the first study who investigate this topic
3. The Introduction section is OK. It would be better if you mentioned why you only examine the TGF-Beta only in this study?
4. It would be better add the reason why you choose the genistein instead of others natural active compound?

 
Material and Methods:

1. It would better if you add the detail protocol or cite  the protocol reference how to make or obtain the genistein
2. It would better if you add the reason why you choose 50 gram force to deliver the orthodontics tooth movement? please state what kind of force 50gf (light force or heavy force).
3. Please add the detail about the healthy condition of animal model, how to examine the condition of animal model? did you get a help from veterinarian? please state
4. Please explain how to purify the isolated GCF from animal study? how to minimize the GCF contamination and the bias?
5. The author mentioned "During GCF collection all animals were sedated" please add the detail protocol how you sedated the animal study.
6. In this study, the author perfomed the ANOVA and  Tukey HSD. It would be better if the author also describe the Levene's and Shapiro Wilk test.

Results:

1. It would be better to understand and attract the reader if you present the data in the diagrams.
2. Please add the result of Levene's and Shapiro Wilk test.
3. Please indicate whether the difference is significant or not in your graph/ diagrams as well, using some symbols (asteric).

 
Discussion:

1. Discussion of the results is quite comprehensive. In analyzing the results, the authors also show citations from the previous study to support the explanation of these results.
2. The answer to the hypothesis of this study have been included at the beginning of the discussion section.
3. Please mention the limitation of this study in the discussion section.

References:

 The supporting references are OK and adequate.

Is the work clearly and accurately presented and does it cite the current literature?

Yes

If applicable, is the statistical analysis and its interpretation appropriate?

Yes

Are all the source data underlying the results available to ensure full reproducibility?

Yes

Is the study design appropriate and is the work technically sound?

Yes

Are the conclusions drawn adequately supported by the results?

Yes

Are sufficient details of methods and analysis provided to allow replication by others?

Yes

Reviewer Expertise:

Orthodontic Research (Orthodontic Tooth Movement)

We confirm that we have read this submission and believe that we have an appropriate level of expertise to confirm that it is of an acceptable scientific standard.

# Response

**Published:** 2020-05-29

**Authors:** Ananto Ali Alhasyimi

**Affiliations:** Universitas Gadjah Mada, Indonesia

Dear Prof **Narmada and Dr Nugraha**

 Thank you for your kind assistance in reviewing our manuscript and providing us with valuable advice for the manuscript and our future work. We have been working throughout the review.

 We have added this information in the present manuscript. We thank you again for the valuable insight.
